# Development of Pericarditis Following Implantation of Micra Leadless Pacemaker

**DOI:** 10.7759/cureus.70027

**Published:** 2024-09-23

**Authors:** Anita M Medepalli, Blake T Edwards, Harry Eyituoyo, Pooja Patel, David C Parish

**Affiliations:** 1 Internal Medicine, Mercer University School of Medicine, Macon, USA; 2 Internal Medicine, Atrium Health Navicent Medical Center, Macon, USA

**Keywords:** complications to pacemaker implantation, heart block, leadless pacemakers, pacemaker, pericarditis, second degree heart block

## Abstract

Pacemakers are frequently essential in managing conduction disorders. Recently, the advancement of leadless pacemakers (LPs) has emerged, offering an alternative without the need for an upper shoulder incision or pacing leads. Despite the advantages, including reduced infection risk and improved durability, the novel nature of LPs means that some complications are still being identified. This report presents a unique case of pericarditis occurring after the implantation of a Micra leadless pacemaker (MLP) (Medtronic, Minneapolis, MN). It demonstrates the critical need for careful post-procedural monitoring to promptly detect and address potential complications associated with this emerging technology.

## Introduction

Pacemakers have long been used in the management of conduction disorders. Leadless pacemakers (LPs) have recently come to the forefront as an attractive option for patients. LPs demonstrate several potential benefits, including reduced infection risk and improved durability [1].

This case report describes a unique and noteworthy complication of pericarditis following the successful implantation of an LP. Through this case report, we aim to contribute to the growing body of knowledge on LPs and demonstrate the importance of comprehensive monitoring of patients following the procedure to identify and manage potential complications.

## Case presentation

**Figure 1:**
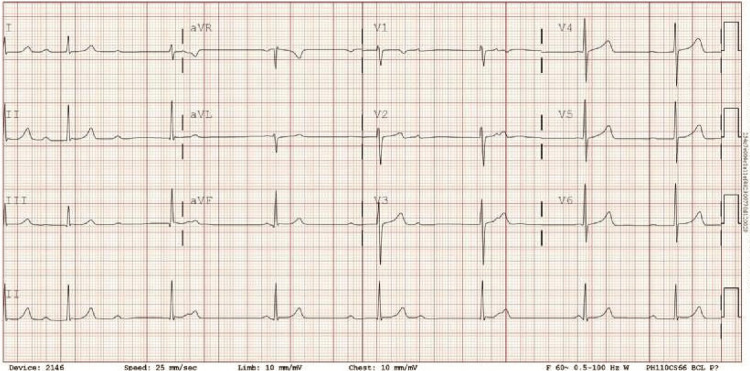
EKG on day 1 of admission, which demonstrated a high-grade, type II second-degree heart block. The patient was referred to the electrophysiologist, who advised for leadless pacemaker placement.

A 29-year-old female presented to the Atrium Health Emergency Center (day 1 of admission) with complaints of shortness of breath, cough, and weakness. Physical examination revealed bilateral lower extremity edema, bradycardia (heart rate in the 50s), and an irregular rhythm. A cardiologist was consulted and diagnosed the patient with symptomatic bradycardia secondary to high-grade, type II second-degree atrioventricular (AV) block (Figure 1). This was intermittent, and no reversible causes were identified (such as structural heart disease, electrolyte, thyroid abnormalities, or connective tissue disorders) [2]. The patient was subsequently referred for implantation of the Medtronic leadless Micra AV2 pacemaker (MC2AVR1) (Medtronic, Minneapolis, MN) in VDD (ventricular paced, dual chamber (atrium and ventricle) sensed, dual response to a sensed beat (either inhibition or tracking) in response to a sensed beat) pacing mode rather than a traditional pacemaker, due to their homelessness, which made consistent attendance at follow-up appointments challenging.

**Figure 2:**
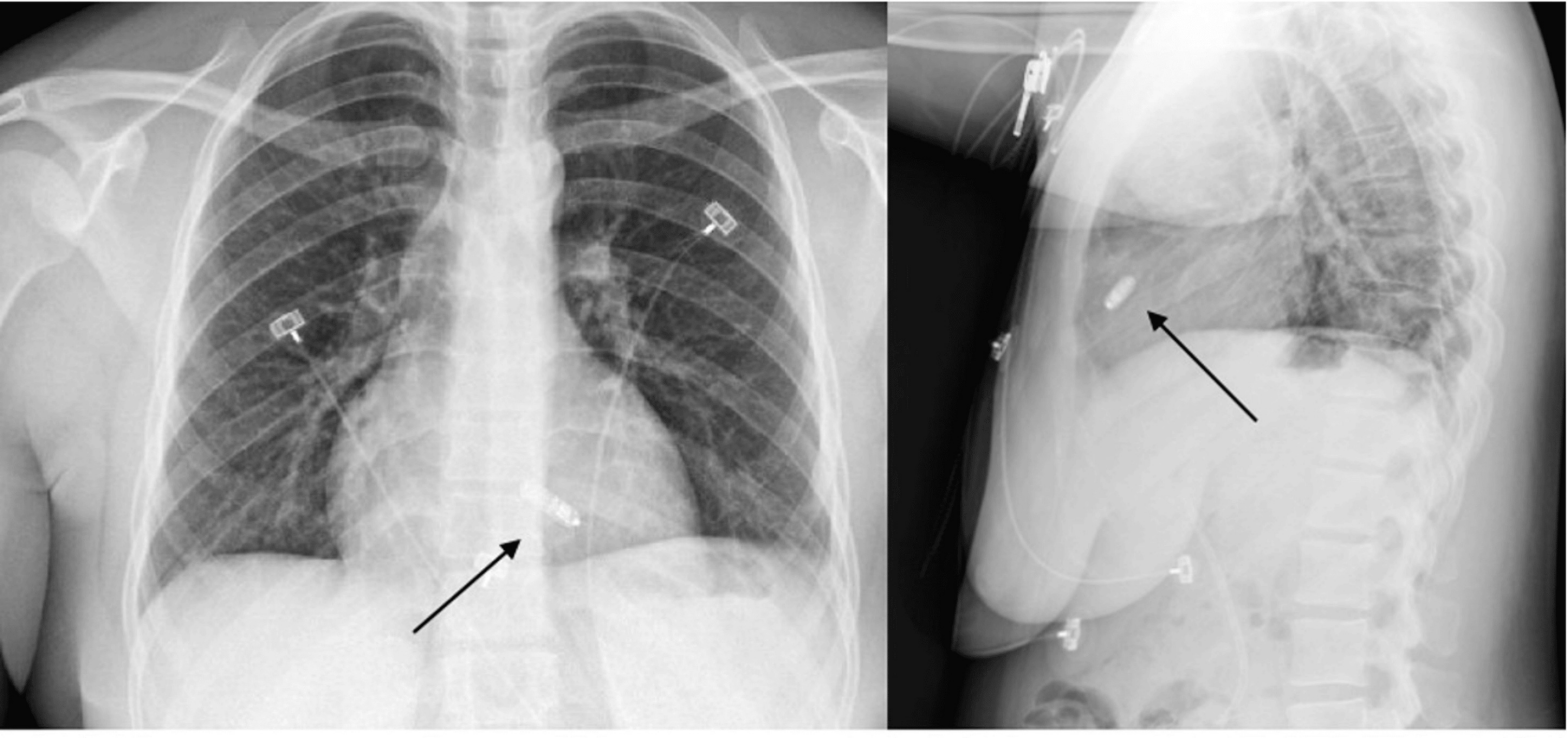
Chest X-ray following pacemaker implantation (arrows) demonstrating appropriate placement.

On the fourth day of admission, the patient underwent a pacemaker implantation procedure. The patient was sedated with fentanyl and midazolam and continuously monitored with pulse oximetry, blood pressure, and electrocardiogram (EKG) throughout the 30-minute procedure. Both the right and left femoral areas were prepped and draped in a sterile manner, prophylactic intravenous antibiotics were administered, and local anesthesia was applied to the subcutaneous tissues. The right femoral vein was accessed using an 18-gauge Cook Needle (Cook Medical, Bloomington, IN) under fluoroscopic guidance, with the guidewire confirmed in the vein via fluoroscopy. Progressive dilation was performed at the access site, followed by the insertion of a 24 Fr sheath (Cook Medical, Bloomington, IN) into the right atrium under fluoroscopic guidance. An Amplatz guidewire (Boston Scientific, Marlborough, MA) was advanced into the superior vena cava, and the introducer delivery system was positioned from the inferior vena cava to the mid-right atrium. After removing the sheath introducer, the catheter was flushed, and a 3000-unit heparin bolus was administered to prevent thrombosis. The Micra leadless pacemaker (MLP) (Medtronic, Minneapolis, MN) guide system was prepared, flushed with saline, and advanced to the septal position of the right ventricle apical septum under fluoroscopic guidance. After engaging the septal wall, the contrast was injected to identify trabeculation, and the MLP was successfully delivered to the apical septum, with the tines confirmed to be adequately flared during the tug test, indicating successful fixation. The post-procedural chest X-ray confirmed appropriate pacemaker placement (Figure 2). Routine post-operative care, including antibiotics, was administered.

**Figure 3:**
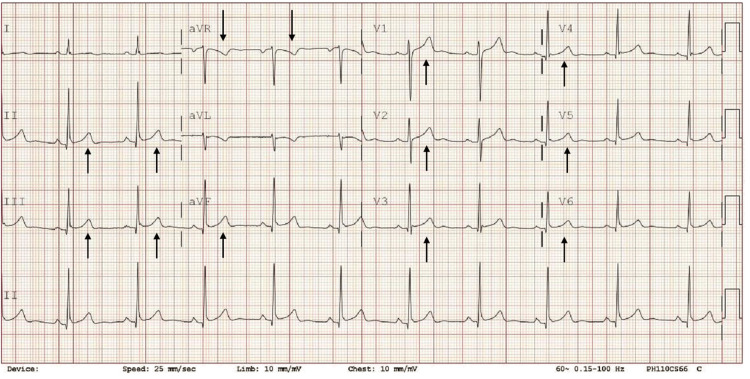
EKG on day 5 of admission (morning after the procedure), which demonstrated pericarditis complication (arrow pointing to ST-elevation).

**Figure 4:**
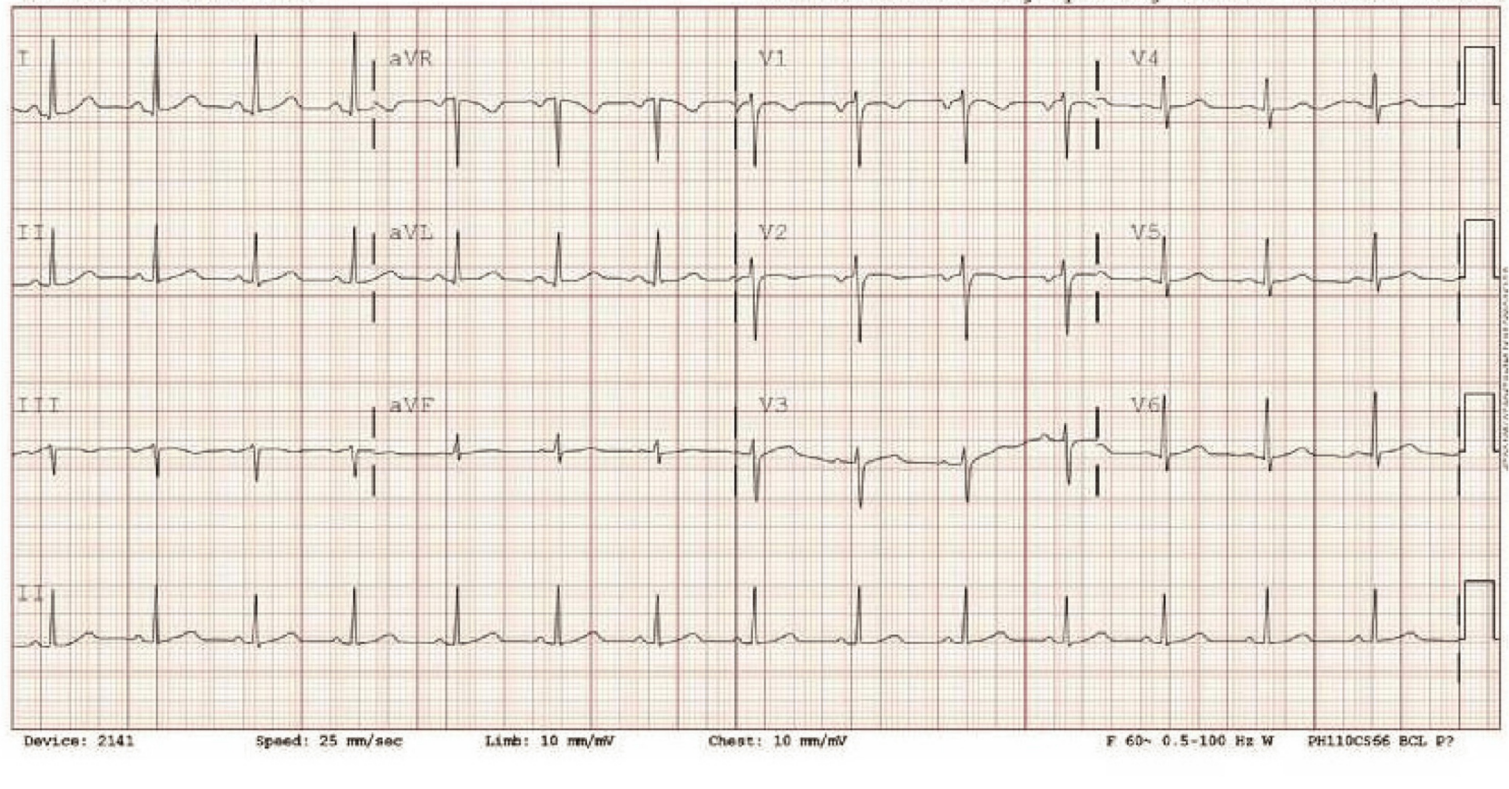
EKG on day 8 of admission (discharge date), which demonstrated resolution of pericarditis.

The following morning, day 5 of admission, the patient reported chest pain, which was relieved by acetaminophen, and chest wall tenderness. Laboratory tests revealed elevated high-sensitivity (hs) troponin levels: 1743 ng/L on the same day, increasing to 2227 ng/L, compared to <4 ng/L prior to the procedure. Erythrocyte sedimentation rate (ESR) was elevated at 28, but C-reactive protein (CRP), thyroid stimulating hormone (TSH), T4, Coxsackie titer, Rh factor antibodies, cyclic citrullinated peptide (CCP) antibodies, double-stranded DNA antibodies (dsDNA), and antinuclear antibodies (ANA) tests were negative. A 12-lead EKG demonstrated diffuse ST-elevation (Figure 3). The patient was treated with oral colchicine, starting with 1.2 mg stat, then 0.6 mg twice a day for two weeks. Symptoms resolved, and by day 8 of admission (discharge date), the repeat hs troponin level had decreased to 503 ng/L, with a repeat EKG showing resolution of ST-elevation (Figure 4).

## Discussion

Pacemakers have long been used to treat arrhythmia and other conduction-related disorders. Pacemakers are traditionally inserted via a surgical incision and utilize leads. Recently, LPs have risen in popularity due to their small size and reduced risk of complications. 

LPs are implanted directly into the heart via a catheter inserted through the femoral vein, eliminating the need for a chest incision and a device pocket. Since there is no incision, this approach reduces the risk of infection, particularly pocket infections and lead-related endocarditis, compared to traditional pacemakers [1]. Additionally, LPs are less prone to tine dislodgment compared to traditional pacemaker’s lead dislodgement, thus offering a more durable solution for patients who may have difficulties with continuous follow-up appointments and monitoring [3].

Despite these advantages, the relatively recent introduction of LPs means that many of their potential complications are still being elucidated. Known risks associated with LP implantation include cardiac perforation, pericardial effusion, cardiac tamponade, embolism and thrombosis, ventricular arrhythmias, pneumothorax, and issues related to the groin puncture site, such as arteriovenous fistulas [4]. While endocarditis is less common with leadless devices, it remains a possible complication [5]. Although pericarditis is not a major device- or procedure-related complication [6], it is a clinically relevant non-major complication that can delay discharge and necessitate additional medication for pain and inflammation control. Clinicians should be most alert to inflammatory complications like pericarditis in patients with recent viral infections, cardiac procedures, autoimmune conditions, trauma, or certain medications. Early detection protocols include routine ECGs, echocardiography, inflammatory marker tracking, patient education for symptom monitoring, and imaging when clinically indicated [7].

In this case, the development of pericarditis could be attributed to the MLP’s long tines embedding into the cardiac RV apical septal tissue. These tines could have generated localized inflammation and swelling within the septal endocardium and myocardium. This inflammation may have extended to the pericardium [8]. The inflammation of all three layers could have contributed to the elevated hs troponin levels and the pericarditis observed in this patient.

## Conclusions

This case highlights a rare but important complication of pericarditis following the implantation of an LP. It demonstrates the need for thorough post-procedural monitoring and to quickly identify and address potential inflammatory responses associated with LP implantation.

Despite the occurrence of complications, LPs offer several advantages over traditional pacemakers, including a reduced risk of infection and fewer issues related to lead dislodgment. These benefits make LPs a valuable option in the management of patients requiring pacing therapy. However, continued research and reporting of clinical experience remains essential to fully understand and mitigate the potential risks of these innovative devices.
